# Competition and training load of high-level competitive non-motorised surface water sports athletes: a scoping review of athlete monitoring methodology

**DOI:** 10.3389/fspor.2026.1811408

**Published:** 2026-07-28

**Authors:** Claire Nancy Walker, Michael Lambert, Karen Estelle Welman

**Affiliations:** 1 Division of Movement Science and Exercise Therapy, Department of Exercise, Sport and Lifestyle Medicine, Faculty of Medicine and Health Science, Stellenbosch University, Stellenbosch, South Africa; 2 Division of Physiological Science and Health Through Physical Activity, Lifestyle and Sport, Department of Human Biology, Faculty of Health Sciences, University of Cape Town, Cape Town, South Africa

**Keywords:** athlete monitoring, competition load, non-motorised surface water sport, scoping review, training load

## Abstract

**Introduction:**

Monitoring athletes during training and competition is necessary to gather information on athletes' fitness and fatigue to prescribe training loads more precisely. The goal of this process is to improve performance and reduce injury risk. This scoping review aimed to examine and map existing research on monitoring methods for high-level athletes in non-motorised surface water sports, thereby identifying the most commonly used method(s) in these competitive sports and guiding future research.

**Methods:**

The Joanna Briggs Institute's scoping review framework served as the guide to provide clarity and rigour in the review. Studies included in this review were published in English in peer-reviewed journals and were identified through a systematic search of four databases: Academic Search Premier, Google Scholar, SPORTDiscus and SpringerLink. The final search of these databases was conducted in August 2025.

**Results:**

Twenty-two papers were deemed relevant for inclusion in the final review. Methodologically, the literature converged on heart rate-based internal load metrics (71%, 15/21) and GPS-derived external load metrics (47%, 8/19), however these are imported from land- and ergometer-based environments and are not yet validated against the environmental load that defines non-motorised surface water sports.

**Discussion:**

Practically, until environment-adjusted metrics exist, all metrics should be interpreted within environmental contexts. Furthermore, the review highlights the need to increase the use of athlete monitoring methods, particularly for youth and females, and in sports such as sailing and canoeing. Future research should aim to develop ecologically valid monitoring methods in a range of non-motorised surface water sports.

## 1 Introduction

Identifying competition and training demands is essential for designing appropriate training programs that maximise athletic performance (1, 2). Consequently, appropriate athlete monitoring processes are necessary to manage these demands and support optimal development (3, 4). This monitoring process allows sport scientists to quantify workload, i.e., demands an athlete is exposed to during competition and training, alongside the athlete's subsequent responses (5, 6). Fundamentally, these loads are calculated as the product of activity duration and intensity.

Tracking loads is central to an athlete's ultimate level of performance, as research supports a positive relationship between athletes' loads and injury rates (7). Consequently, as the demands on competitive athletes increase, continual monitoring of individual responses to training stimuli has become crucial in training setups. When an athlete reaches the fatigue threshold, it will deplete their reserve capacities and lead to a failure to adapt. In return, this may result in disproportionately accumulated fatigue and could lead to overtraining (8). Thus, athlete monitoring methods need to determine an athlete's tolerance to the training stimulus and whether they should maintain, increase or decrease their current load. The rationale for this practice is based on Selye's General Adaptation Syndrome, which states that an athlete will adapt to stressors they might experience to meet their demands (9). Whereas competition load gives insight into the physical and physiological demands of sports competition, which is critical to determine if training is adequate for the specific requirements of the competition (1).

Athlete monitoring can be organised based on a four-tier framework that categorises athlete monitoring into a system (organisational data management platform), a tool (data collection instrument), a load metric (specific data variable), and an athlete's internal response (physiological or psychological reaction) to the imposed load. Further to this, athlete monitoring approaches can be divided at a macro-level into (i) tracking [e.g., global positioning systems (GPS)], (ii) self-report [e.g., ratings of perceived exertion (RPE)] or (iii) performance testing (e.g., submaximal or maximal tests). Notably, athlete monitoring often takes the form of indirect measures of maximal performance or other relevant sport-specific characteristics. Moreover, research on load monitoring is typically divided into two categories; either (i) external load [e.g., time, training frequency, distance, video-based time-motion analysis (TMA), power output, speed, acceleration and neuromuscular performance tests]; or (ii) internal load (e.g., RPE, heart rate (HR), blood lactate (La), oxygen uptake, wellness questionnaires, and biochemical, hormonal and immunological responses) (10). According to Wallace and colleagues (11), external load is the work done by the athlete, independent of their internal characteristics or strain. In contrast, internal load is more about the body's response to the external load (i.e., relative physiological and psychological stress inflicted on the athlete) (12). Therefore, external load provides insight into the work completed and the athlete's capabilities, while internal load is often used to determine fitness outcomes by shaping the load and subsequent adaptation. In addition, the ratio between external and internal loads has been used to assess an athlete's fatigue state (12).

In this review, non-motorised (specifically environmentally and self-propelled) surface water sports are defined as a sport involving the control and propulsion of an object (e.g., a boat or board) over the surface of the water, using either environmental factors (i.e., wind and/or waves) or self-propelled methods (i.e., paddling). Examples of sports include kayaking, kiteboarding/surfing, rowing, sailing, stand up paddling, and surfing. Comprehensive knowledge of current trends and monitoring techniques in non-motorised surface water sports is crucial for a better understanding of the loads these athletes are exposed to during both training and competition. Particularly since these sports have an added variable to contend with and overcome, namely the “playing” surface or water state. Specifically, to perform optimally, the “playing” surface, which is continuously moving (i.e., with currents or waves) or merely the changeable quality of water (i.e., fluidity) needs to be conquered. The non-motorised surface water sports can be classified by this environmental constraint, distinguishing between predictable, flat-water settings, such as lakes, and highly unpredictable, open-ocean environments. Importantly, in the context of sports, the external load applied to the athlete is heavily influenced by environmental variables. These external forces alter the athlete's mechanical efficiency, meaning that a standard metric, such as speed or distance, cannot be interpreted accurately without accounting for environmental context. Furthermore, changes in environmental variables can significantly affect athletes' biomechanics during sport performance, as shown by Chicoy and Encarnación-Martínez (13) in their analysis of the hiking technique in dinghy sailing.

While athlete monitoring has been well established and is utilised in a range of sporting codes, from team (2, 5, 14–16) to individual sports (17–19), it has not been researched extensively in non-motorised surface water sports. The reason for this may be that the monitoring methods used in land-based sports were initially not deemed feasible for water-based sports. However, this is changing as these sports are becoming more popular, professional, and competitive. Nevertheless, due to the competing athlete's dependence on environmental conditions as well as the actions of the competitors, the competition results in these sports are inherently difficult to analyse (20). Consequently, water-based monitoring often faces additional logistical challenges because the technical equipment used in land-based sports requires extensive modification before it can be successfully implemented in these environments. Furthermore, some of these sports, such as sailing and rowing, take place over vast distances, making it difficult to track/video during the entire event.

To date, two studies have focused explicitly on load monitoring in non-motorised surface water sports. Farley and colleagues (21) explored the use of video-based TMA, GPS devices and HR indices during a surfing competition. Mäestu and colleagues (20) considered performance and training monitoring in rowing, with a specific focus on preparation leading up to a competition. The researchers also suggest that the few studies which have investigated monitoring in rowing have not determined a single marker of load monitoring. While a review by Shepard and Shek (22) did include a brief section on monitoring training of rowers, but the specific focus of this review was on rowing injuries. Whereas the review by Smith and Hopkins (23), studied the measures of rowing performances and the associated performance testing available for rowers. Researchers must start exploring feasible monitoring methods specific to surface water sports rather than those based on other water sports, such as swimming. Accordingly, given that surface water sports are largely under-represented in the literature of athlete monitoring, the purpose of this scoping review is to investigate the existing research surrounding the load monitoring methods implemented by coaches, sports scientists and researchers, as well as identify gaps in the knowledge base of monitoring load in non-motorised surface water sports specifically for high-level competitive athletes (including national and international level athletes).

Therefore, the aims of this scoping review were to (i) identify and characterise the main methodologies used in monitoring training and competition load characteristics (i.e., external and internal) in high-level competitive non-motorised surface water sports athletes with the goal of determining which monitoring method is the most commonly used to quantify workload, and (ii) to provide future directions for research on athlete load monitoring in these sports.

## 2 Methods

### 2.1 Study selection: inclusion and exclusion criteria

The proposed scoping review was conducted in accordance with the Joanna Briggs Institute methodology for scoping reviews (24) and is reported in accordance with the PRISMA-ScR checklist (25). A review protocol was not developed or registered for this scoping review prior to the commencement of the study. All descriptive observational study designs considered for this review were published before 01 August 2025 in English and were available in full text and peer-reviewed in scientific journals (see Table 1 for complete inclusion and exclusion criteria). No limits were placed on the year of publication.

**Table 1:** Scoping review specific in- and exclusion criteria for publications.

Inclusion criteria	Exclusion criteria
Surface water sports^a^ [i.e., canoeing, canoe polo, kayaking, kiteboarding, rowing, sailing, stand up paddling, surfing and windsurfing]. Any age and sex. Any form of athlete monitoring [i.e., self-report, tracking or performance testing] during a training session and/or formal competitive event. Monitoring measures [i.e., internal TL—HR, HR recovery, RPE, session RPE, neuromuscular function, biochemical/ hormonal/ immunological assessments, questionnaires (e.g., REST-Q), sleep quantity & quality; external TL—GPS, video-based TMA, session duration & frequency, repeat efforts, speed, accelerations, power output, training type, distance]. 80% of the sample must be surface water sport^a^, high-level competitive, athletes. peer-reviewed observational studies (including both descriptive & analytical designs).	Only provided a technical description of the movements in the sport. Review articles, single-participant case reports, epidemiological studies and interventions. Not full-length papers or grey literature (such as conference proceedings) and non-peer-reviewed papers (like dissertations and theses). non-competitive or leisure sports, i.e., recreational, amateur and club level athletes

a Non-motorised i.e., environmentally & self-powered; TL, training load; HR, heart rate; RPE, ratings of perceived exertion; REST-Q, recovery-stress questionnaire; GPS, global positioning systems; TMA, time-motion analysis.

Participants in the respective studies were limited to athletes classified as Tier 3 (Highly Trained/National Level) or Tier 4 (Elite/International Level) according to the framework established by McKay and colleagues (26). Because most included studies (published 1999–2021) predated the McKay and colleagues (26) framework and used heterogeneous descriptors (e.g., “national”, “international”, “elite”), athletes were classified retrospectively from the competition level reported in each study. Athletes described as competing at national level (e.g., national championships or national squads) were assigned to Tier 3 (Highly Trained/National Level), whereas those described as competing internationally (e.g., World Cup, World Championship or Olympic competition) were assigned to Tier 4 (Elite/International Level); where a study reported a mixed cohort (e.g., “national and international”), the tier was assigned according to the highest competition level at which the reported sample competed. Two reviewers (CW, KW) applied this mapping independently, with disagreements resolved by a third reviewer (ML). This classification ensures a standardised, comparable population of athletes engaged in intense, systematic training for high-level competition. The reason high-level competitive athletes were chosen as the study population was to ensure that participants in the studies competed in similar events.

Publications included were required to describe the methodology and metrics used to monitor the athletes during training and/or competition of non-motorised surface water sports.

### 2.2 Data sources and search strategies

**Figure 1:**
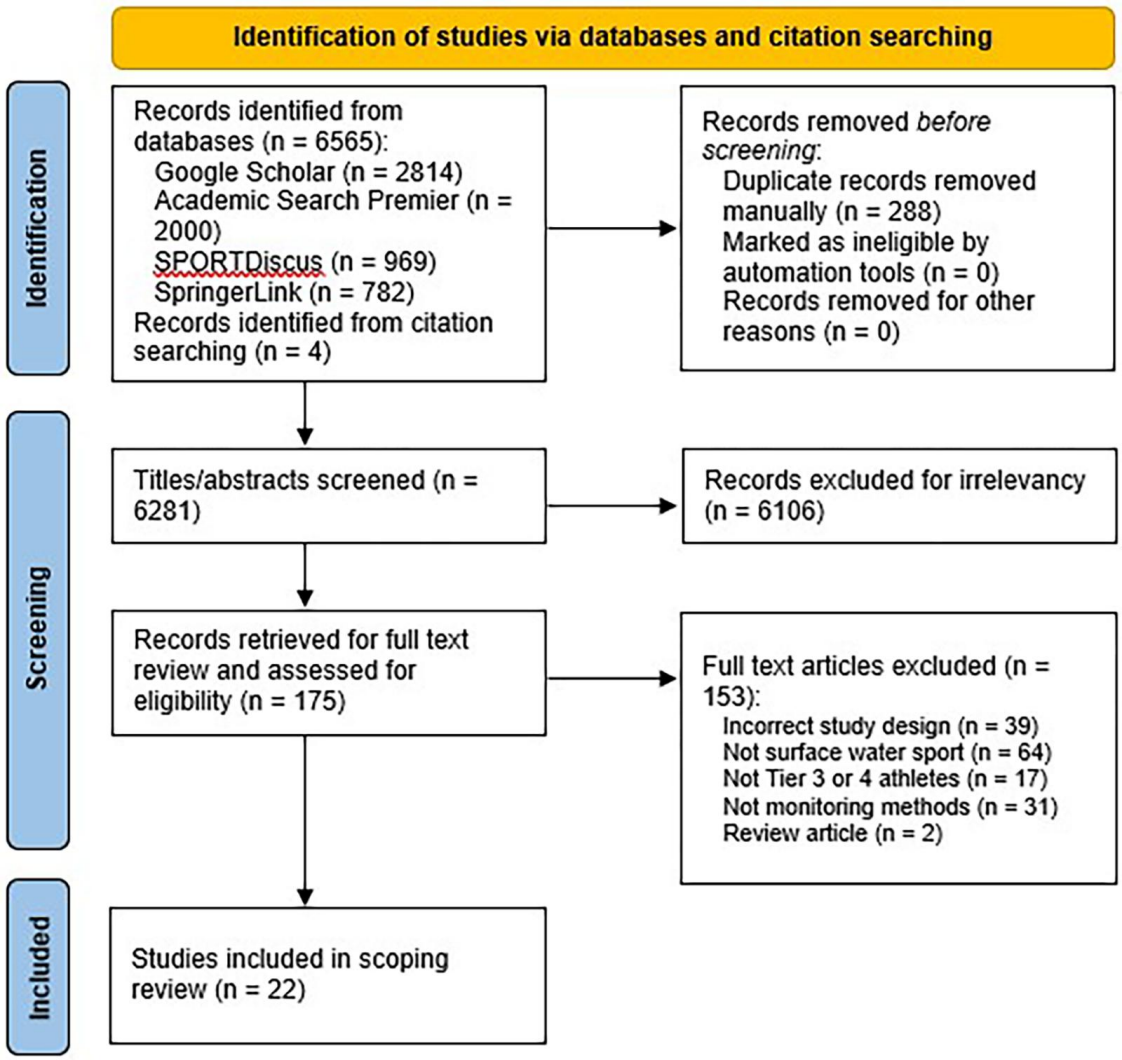
PRISMA-ScR diagram of search results and article screening (24).

The search strategy aimed to locate published studies in English only. A three-step search strategy was utilised in this review. First, an initial limited search of Google Scholar was conducted to identify articles on the topic. The text words contained in the titles and abstracts of relevant articles, and the index terms used to describe the articles were used to develop a full search strategy for an additional three databases, specifically Academic Search Premier; Google Scholar, SPORTDiscus™ and SpringerLink (Supplementary Appendix 1). The search strategy, including all identified keywords and index terms, was adapted for each included database. Finally, the reference list of all included studies were screened for additional studies. The final search was conducted in August 2025. The process used for selecting the articles used in this review is outlined in Figure 1.

Following the search, all identified citations were collated and uploaded into EndNote Version 22, 2025 (Clarivate Analytics, PA, USA), and duplicates were removed. Two reviewers (CW, KW) screened independently at title/abstract and full-text stages; inter-rater reliability was not formally quantified, instead disagreements were resolved through discussion by a third reviewer (ML). The percentage agreement was not calculated at the time. Consistent with PRISMA-ScR, formal critical appraisal is optional for scoping reviews; we nonetheless report a methodological-quality assessment (Table 3) to inform interpretation, not eligibility.

### 2.3 Data extraction

Data was extracted from papers included in the scoping review by two independent reviewers using a data extraction tool developed by the reviewers. For calibration purposes, the data extraction tool was piloted on five studies before full charting. Data on athlete characteristics (sex, age, level of competition, training phase), the length of the monitoring period, the training phase, and the monitoring method used (self-reported, tracking, performance testing), as well as all monitoring outcome variables, were collected independently by two reviewers (CW, KW). Data extraction accuracy was independently verified by the third reviewer (ML).

### 2.4 Risk of bias assessment

An assessment of the methodological strength and risk of bias for the eligible studies was determined using the National Institutes of Health (NIH) Quality Assessment Tool for Observational Cohort and Cross-Sectional Studies scale (27), which ranges from 0 to 14 points. A score of over 80% was considered “good”, 60%–80% “fair”, and under 60% “poor”. In general, a “good” study has the least risk of bias, and its results are considered valid. The broad “fair” category is susceptible to bias that is not sufficient to invalidate its results, but studies with this rating will vary in their strengths and weaknesses. While a “poor” rating indicates a significant risk of bias. Due to the limited studies, available studies rated poor were not excluded. Scores were adjusted for descriptive vs. analytical studies (28). Specifically, if the question did not apply to a descriptive, non-analytical study, it was given an “NA” and did not count negatively towards the quality rating. The quality appraisal was done by two reviewers (CW, KW), and if any disagreements were noted a third reviewer was consulted (ML). The overall agreement (Good/Fair/Poor) among reviewers was high (23/27 = 85%).

## 3 Results

### 3.1 Main findings

The initial search yielded 6,569 potentially relevant articles. After duplicate removal, 6,281 articles were screened for titles and abstracts. Following this, 175 full-text articles were retrieved for full-text review, and of these, 22 studies met the inclusion criteria. A summary of this process is presented in Figure 1.

### 3.2 Characteristics of the studies

Most studies were considered correlational, analytical, observational studies in naturalistic settings. An overview of the articles included is provided in Table 2. The articles included from the respective sports were canoeing (*n* = 0), canoe polo (*n* = 1), kayaking (*n* = 3), kiteboarding (*n* = 1), rowing (*n* = 9), sailing (*n* = 0), stand-up paddling (*n* = 1), surfing (*n* = 4) and windsurfing (*n* = 3). There has been a slow growth in the number of studies published on this topic; 73% of the studies included were published after 2010, and of these, just over half (56%) were published after 2015. While 27% were published between 1999 and 2009. Ten studies only focused on describing load during competition, while five studies monitored a combination of load during training sessions and competition; one study only monitored load in the recovery phase, while the remaining six studies only observed load over the preparatory and overload phases. For all included studies, the timeframe varied from a single session up to 42 weeks.

**Table 2:** Overview of studies included in the scoping review.

Authors	Surface water sport	Sample size (*n* (sex))	Age (years) $\left(\bar{x}\pm \mathrm{SD}\right)$	Level of competition	Phase (Competition/Training)	Monitoring Period	Monitoring approach			Outcome variables/Metrics	Training load category	
							Self-reported	Track-ing	Perform-ance testing		Int.	Ext.
Forbes et al. (35)	Canoe polo	8 (M)	25 ± 1	International	Competition phase	Single session (championship)		X	X	HR indices, VT, VO2 indices, TMA, anaerobic power, strength (grip), flexibility (sit & reach)	X	X
Kenttä et al. (49)	Kayaking	11 (6M:5F)	20 ± 1	Elite	Preparatory phase	3 weeks (training camp)	X		X	RPE, POMS, maximal strength (chins, dips, abs, bench press, bench pull), duration, sport-specific kayak performance test	X	X
Garatachea et al. (31)	Kayaking	8 (5M:3F)	17 ± 2	International	All	42 weeks	X		X	RPE, POMS, RESTQ-sport, blood samples, maximal strength (bench press)	X	X
Borges et al. (32)	Kayaking	10 (6M:4F)	17 ± 1	International	Recovery phase	7 weeks	X	X	X	HR indices, blood samples [La], sRPE, TRIMP, iTRIMP, VO2 indices, MAP, duration, distance, speed, on-water time trials	X	X
Caimmi & Semprini (50)	Kiteboarding	5 (M)	34 ± 5	National	Competitive phase	Single session (regatta series)		X	X	HR indices, distance, speed & direction, frequency & duration of each beat, wind angle	X	X
Fiskerstrand & Seiler (29)	Rowing	21 (M)	CD	International	All	30 years	X		X	VO_2_ indices, blood samples, training volume, training type, rowing performance test	X	X
Messonnier et al. (51)	Rowing	21 (M)	22 ± 3	International & national	Preparatory phase	Single session (7–13 sessions)	X		X	HR indices, blood samples [La], EE, duration, PAQAP questionnaire	X	X
Guellich et al. (48)	Rowing	36 (M)	19 ± 1	International & national	All	37 weeks			X	HR indices, blood samples [La], VO_2_ indices, duration, stroke frequency	X	X
Smith et al. (52)	Rowing	20 (10M:10F)	M – 24 (21–30); F – 23 (19–31)	International (elite)	NR (Overload phase)	4 weeks x 2 (x 7 years)	X		X	Wellness questionnaire, blood samples [La], saliva samples, power output, training volume	X	X
Plews et al. (30)	Rowing	9 (5M:4F)	NR	International (elite)	Preparatory phase	26 weeks			X	HR indices, blood samples [La], duration	X	
Tran et al. (34)	Rowing	14 (12M:2F)	26 ± 3	International (elite)	Specific preparation phase	4 weeks	X		X	HR indices, sRPE, iTRIMP, TRIMP, VO_2_, VCO2, VT, RCP, T2minute, duration	X	X
Tran et al. (36)	Rowing	21 (14M:7F)	M – 28 ± 3; F – 32 ± 3	International (elite)	Specific preparatory & Competitive phase	6 months		X	X	VO_2_, blood samples [La], T2minute, power output, weekly training frequency, duration, distance	X	X
Plews et al. (19)	Rowing	4 (1M: 3F)	NR	International	Preparatory & Competitive phase	62 days		X	X	HR indices, distance, speed	X	X
Watts et al. (53)	Rowing	16 (8M:8F)	19.3 ± 1.1	National	NR	4 months		X	X	HR indices, velocity, stroke rate	X	X
Schram et al. (54)	Stand up paddleboard	10 (6M:4F)	NR	National	Competitive phase	Single session (race)		X	X	HR indices, duration, speed, route/ distance	X	X
Farley et al. (21)	Surfing	12 (M)	24 ± 6	National	Competitive phase	Single session (competition heat)		X	X	HR indices, distance, TMA, duration, number of paddling bouts, speed	X	X
Farley et al. (45)	Surfing	41 (M)	23 ± 6	National	Competitive phase	Single session (competition series)		X		Distance, speed, duration, TMA		X
Fernández-Gamboa et al. (42)	Surfing	10 (M)	29 ± 11	National	Competitive phase	Single session (competition heat)	X	X	X	Distance (paddling & wave riding), percentage duration, velocity, RPE	X	X
Silva et al. (33)	Surfing	12 (6 M: 6F)	16 ± 1	National & regional	Competitive	Single session (competition heat)		X	X	HR indices, RPE, sRPE, GPS (distance, speed & pace), duration	X	X
Guevel (46)	Windsurfing	8 (M)	23 ± 3	International & national	Competitive phase	Single session (3 competitive regattas)		X	X	HR indices, blood samples [La], duration, wind intensity	X	
Chamari et al. (47)	Windsurfing	10 (9M:1F)	21 ± 4	International	Competitive phase	Single session (competitive regatta)			X	HR indices, VO_2_ indices, blood samples [La], wind intensity, duration	X	
Pérez-Turpin et al. (55)	Windsurfing	45 (M)	30 ± 10	National	Competitive phase	Single session (race)		X	X	HR indices, speed, route/ distance	X	X

[La], blood lactate; CD, cannot determine; EE, energy expenditure; Ext., external; HR, heart rate; Int., internal; iTRIMP, individual training impulse; M, male; F, Female; MAP, mean aerobic power; NR, not reported; PAQAP, physical activity questionnaire; POMS, Profile of mood state; RCP, respiratory compensatory point; RPE, rate of perceived exertion; sRPE, session-RPE; T2minute, time-in-zone approach; TMA, time-motion analysis; TRIMP, training impulse; VO_2_, oxygen uptake; VT, ventilatory threshold.

### 3.3 Characteristics of the athletes

Although almost half the studies (*n* = 11) included a mixture of both sexes, of the 352 athletes, 84% were male. The remaining 16% of females included participated in kayaking (*n* = 3 studies), rowing (*n* = 6 studies), and one each in stand-up paddling, surfing, and windsurfing. No study looked at only female athletes. The ages of the included participants ranged from 17 to 34 years. Three studies did not provide a clear indication of the ages of the athletes included (19, 29, 30). While only three studies (31–33), two in kayaking and one in surfing, reported monitoring of youth athletes (≤18 years).

### 3.4 Monitoring methods

The monitoring methods investigated were categorised as self-report, tracking and performance testing. Tracking considers aspects of external load measured through GPS (such as distance and frequency of event) or video-based TMA (for example, percentage time spent in sport mode, i.e., paddling in surfing) (34). In this review, only two studies described loads during training and competitions across all three monitoring approaches; six studies described a combination of self-report and performance-testing measures; and ten studies described a combination of tracking and performance-testing metrics (see Table 2). Three studies described performance testing measures only, while one considered tracking only. Interestingly, 93% (14/15) of the studies that reported loads during competition used some form of performance-testing approach. The included studies also varied in that both internal and external loads were reported by 18 of the studies (82%, 18/22), while three (14%, 3/22) reported only internal loads and one (5%, 1/22) on external loads during competition only.

In this analysis, each percentage is the number of studies reporting that metric divided by the total number of metric reports across all the studies monitoring internal load (*n* = 21) or external load (*n* = 19). The most reported metrics of athlete monitoring for internal load are HR-based indices (71%, 15/21), followed by blood samples (43%, 9/21), VO2 (oxygen uptake) (33%, 7/21) and RPE methods (29%, 6/21). Whereas, for external loads, duration (79%, 15/19) and specific GPS measures including distance (sometimes referred to as course sailed) (47%, 8/19) and speed (47%, 8/19) were most often reported. These were followed by strength testing (16%, 3/19), power output (11%, 2/19), and training volume (11%, 2/19). Other metrics included energy expenditure, questionnaires, training impulse (TRIMP), ventilatory threshold, saliva samples, number of efforts, training types, and reported wind intensities. Two studies examined a novel method for determining load in rowing, specifically the T2minute method (35, 36). Based on these findings there appears to be evidence that HR-based indices, duration and GPS metrics (distance and speed or velocity) are most often used when monitoring loads of athletes in non-motorised surface water sports compared to other metrics.

Within the studies included sporting disciplines can be meaningfully classified based on environmental predictability or lack thereof. We grouped the non-motorised water sports into either structured, more closed environments (e.g., rowing, canoe polo and kayaking) or less predictive, open-water environments (e.g., surfing, windsurfing, kiteboarding, and stand-up paddleboarding). In structured, closed environments, which represent 59% of the literature (13/22), researchers frequently utilised HR-based indices (62%, 8/13), duration (54%, 7/13), and blood samples (54%, 7/13). None of these studies reported environmental constraints, however, 54% (7/13) of the studies presented sport-specific on water performance testing approaches. In contrast, literature addressing less predictive, open-water environments (41%, 9/22) relied mostly on HR-based indices (78%, 7/9), duration (78%, 7/9), and distance (67%, 6/9) metrics. Wind metrics and route or direction of travel were reported in 34% (3/9) and 34% (3/9) of these studies respectively. Notably, none of these monitoring methods accounted for the water surface state itself.

It is worth noting that canoeing and sailing are the only sports for which no studies were found reporting on the loads of high-level athletes. The logistical and technological barriers likely underlying this absence in sailing studies are discussed in the Discussion. All the studies on windsurfing, assumedly the most similar sport to dinghy sailing, reported internal load, particularly HR-based indices, while only one reported external load, specifically distance and speed (measured in competition).

### 3.5 Quality assessment

The results of the quality assessment are presented in Table 3. Most of the studies were of Fair quality (73%), none were of good quality and 27% were of poor quality. Sixty-four per cent of the observational studies were classified as analytical, and the remaining 36% as descriptive observational studies. As a result, questions 6–14 were not considered applicable to the descriptive studies, unless the study included an analytical component. In this case, questions 5 and 14 were considered.

**Table 3:** Results for the quality assessment with quality assessment tool for observational cohort and cross-sectional studies.

Authors	Study design	Q1	Q2	Q3	Q4	Q5	Q6	Q7	Q8	Q9	Q10	Q11	Q12	Q13	Q14	Scoring (%)	Quality rating
Forbes et al. (35)	Descriptive	Y	Y	CD	Y	N	NA	NA	NA	NA	NA	Y	NA	NA	NA	67	Fair
Kenttä et al. (49)	Analytical	Y	Y	CD	Y	N	Y	Y	Y	Y	Y	Y	N	CD	N	64	Fair
Garatachea et al. (31)	Analytical	Y	Y	CD	Y	N	Y	Y	Y	Y	Y	Y	N	Y	N	71	Fair
Borges et al. (32)	Analytical	Y	Y	CD	Y	N	CD	Y	N	Y	Y	Y	N	N	Y	50	Poor
Caimmi & Semprini (50)	Descriptive	Y	Y	CD	Y	N	NA	NA	NA	NA	NA	Y	NA	NA	N	57	Poor
Fiskerstrand & Seiler (29)	Descriptive	Y	Y	Y	Y	N	NA	NA	NA	NA	NA	Y	NA	N	N	63	Fair
Messonnier et al. (51)	Analytical	Y	Y	CD	Y	N	N	N	Y	Y	Y	Y	NA	NA	Y	67	Fair
Guellich et al. (48)	Analytical	Y	Y	Y	Y	N	Y	Y	Y	Y	Y	Y	N	Y	N	79	Fair
Smith et al. (52)	Analytical	Y	Y	CD	Y	Y	N	Y	N	Y	Y	Y	NA	CD	N	62	Fair
Plews et al. (30)	Analytical	Y	N	CD	Y	Y	CD	Y	Y	Y	Y	Y	NA	Y	Y	77	Fair
Plews et al. (19)	Descriptive	Y	N	CD	Y	Y	NA	NA	NA	NA	NA	Y	NA	Y	N	63	Fair
Watts et al. (53)	Analytical	Y	Y	CD	Y	N	CD	Y	Y	Y	Y	Y	NA	Y	N	77	Fair
Tran et al. (34)	Analytical	Y	Y	CD	Y	Y	NA	Y	N	Y	Y	Y	NA	N	N	67	Fair
Tran et al. (36)	Analytical	Y	Y	CD	Y	Y	N	Y	Y	Y	Y	Y	NA	CD	N	69	Fair
Schram et al. (54)	Descriptive	Y	Y	CD	Y	N	NA	NA	NA	NA	NA	Y	NA	Y	N	63	Fair
Farley et al. (21)	Descriptive	Y	Y	N	Y	N	NA	NA	NA	NA	NA	Y	NA	NA	NA	67	Fair
Farley et al. (45)	Descriptive	Y	Y	CD	Y	Y	NA	NA	NA	NA	NA	Y	NA	CD	N	63	Fair
Fernández-Gamboa et al. (42)	Analytical	Y	Y	CD	Y	N	N	N	N	Y	N	Y	NA	NA	N	42	Poor
Silva et al. (33)	Descriptive	Y	Y	CD	Y	N	NA	NA	NA	NA	NA	Y	NA	NA	N	63	Fair
Guevel (46)	Analytical	Y	Y	CD	Y	N	N	N	Y	Y	CD	Y	NA	N	N	46	Poor
Chamari et al. (47)	Analytical	Y	Y	CD	Y	N	N	N	Y	Y	N	Y	NA	NA	CD	50	Poor
Pérez-Turpin et al. (55)	Analytical	Y	Y	Y	Y	N	N	N	N	Y	N	Y	NA	NA	N	50	Poor

Q, question on NIH quality assessment tool for observational cohort and cross-sectional studies; CD, cannot determine; N, no; NA, not applicable; Y, yes.

>80% = Good, 60–80% = Fair, and <60% = Poor; %: Percent.

A limitation of the studies included was small sample sizes (ranging from 4 to 45 participants). Two studies could be considered case studies, but they were included because they met the predefined inclusion criteria (37). Another study by Ballegaard and colleagues (38) was excluded as the authors compared two sailors (one boat) to the average top performers in various international regattas. Conversely, if these studies only include a select population of high-level competitive participants, the small sample sizes are probably admissible. The lack of justification for sample sizes and effect size estimates, as well as the failure to consider potential confounders, were the main methodological shortcomings. The most frequently unreported aspects were “the participation rate of eligible persons” and the loss to follow-up after baseline'. Notably, the strengths included an accurate description of the research question or objectives and clear descriptions of the study population, exposure, and outcome measures.

## 4 Discussion

Athlete monitoring assesses the effectiveness of the training program, minimises the risk of developing non-functional overreaching or injury, as well as providing coaches, sport scientists and support staff with more confidence that they are applying the correct training stimulus for specific athletes (39). This scoping review evaluated the methodologies used to monitor loads of high-level competitive athletes in non-motorised surface water sports. While these non-motorised surface water sports differ significantly in their physiological profiles and environmental demands, grouping them allows for a comprehensive overview of athlete monitoring methodologies across the sports, identifying shared technological challenges and gaps in the literature. Despite a thorough literature search, only 22 studies met the inclusion criteria. The included studies differed in methodology (monitoring methods) and in the number, sex, and age of participants. Studies also differed in the duration of the monitoring period (ranging from a single session to 42 weeks).

Concerning loads for training and competition, the studies which did report HR-based methods, mostly reported on HR-based indices, the training impulse method (TRIMP) (40) or the summated-heart-rate-zones method (41). The utilisation of summated-heart-rate-zones is interesting, considering that the summated-heart-rate-zones method may have face validity but not criterion validity. However, six of the 21 studies which considered internal loads did not report HR-based methods. Heart-rate monitoring appears promising and feasible in these sports; however, as no included study achieved “good” methodological quality, this should be regarded as a provisional recommendation requiring confirmation in higher-quality research.

Some researchers have noted that HR-based methods may cause discomfort (as in surfing) for athletes and have suggested incorporating self-report tools instead (42). The session RPE (sRPE) remains underutilised in non-motorised water sports compared to land or swimming counterparts. Methodologically, the value of sRPE lies in its role as a low-cost, complement to objective metrics such as HR-based methods. Rather than acting as a direct replacement for these methods, sRPE serves as an internal measure of the athlete's subjective load. Importantly, sRPE is strongest when used together with objective metrics like HR-based indices to provide a multi-dimensional cross-check. The validity of this pairing in aquatic environments is supported by Wallace and colleagues (11), who demonstrated that sRPE significantly converges with HR-based internal loads in competitive swimmers (*r* = 0.55–0.94). Highlighting alignment between subjective ratings and physiological stress in these water-based environment. However, using sRPE as a standalone metric potentially creates methodological vulnerability due to conscious bias during self-reporting. As noted by Saw and colleagues (39), athletes may respond in a way they deem to be socially desirable, and as a result, they appear to be coping when in fact they are not or vice versa. Leaning entirely on a self-reported rating leaves coaches blind to these distortions; instead, tracking heart rate and sRPE side by side allows the objective and subjective readings to either confirm or correct each other. Thus, when using self-report methods, it is essential to consider the design of the measure, how the data are collected (e.g., via a mobile application or a hard copy in the form of a book), as well as the individual and situational factors that may influence the athlete.

In terms of external load measures, duration of the session or race was most often observed and reported. While this is likely due to its simplicity, the metric's use as a monitoring tool is inherently limited in surface water sports. This is because identical training durations can yield significantly different physiological loads depending on environmental conditions and the athlete's technical proficiency. For example, weather conditions in rowing significantly affect race times (43). Rather than serving as a standalone proxy for external load, duration is most informative when combined with sport-specific intensity or mechanical measures, for example, stroke rate and power output in rowing, or duration weighted by prevailing wind and water conditions. Integrating duration into such composite metrics, consistent with the load = duration ×  intensity principle, allows it to contribute context rather than carry the full burden of quantifying workload. There has been a consistent increase in the utilization of GPS methods coinciding with the development of technology, both in team and individual sports. The use of these technologies enables a coach or sport scientist to track athletes' real-time loads during training and competition and to quantify their activity profiles. This may be useful when considered with environmental contextual variables in non-motorised surface water sports. Additionally, TMA via video analysis is a method for determining time spent in specific modes of the sport, such as paddling vs. surfing (44, 45), and could be considered a viable athlete-monitoring method in future studies. Thus, while most studies considered distance and speed variables when measuring external load with GPS devices, duration seems to remain a useful determinant of load. A possible explanation for the continued use of duration is that it is one variable a coach has control over among the several uncontrollable factors in these sport types.

Only two studies (both on windsurfing) considered environmental conditions (specifically wind intensity) and their potential effects on the athlete's load (46, 47). Both studies showed that the percentage HR maximum values of windsurfers differ when comparing light (4–8 knots) and moderate (9–13 knots) wind intensities during a competition (46, 47). This is an important distinction to note: in similar sports, there are environmental stressors that should be considered and add to the overall load on athletes. Beyond wind intensity, conditions such as wave height and period, tidal or river flow, and surface state (i.e., glassy vs. choppy water) act as uncontrolled covariates that systematically alter external load. Rather than being acknowledged in passing, these should be instrumented and incorporated into load models to distinguish athlete effort from environmental influences. Notably, even for wind, the only environmental variable studied to date, the evidence is limited; neither windsurfing study examined strong wind (≥16 knots), leaving the upper range of environmental demand uncharacterised. Consequently, comprehensive monitoring methods in these sports should move towards including these unpredictable water conditions to more accurately determine the actual load.

These barriers likely explain the absence of eligible sailing studies noted in the Results. The extent of the search supports this interpretation in that truncated search terms (e.g., sail*, canoe*) captured all variants of the word, no date restriction was applied, and four databases (Academic Search Premier, Google Scholar, SPORTDiscus and SpringerLink) were combined with backward citation searching of all included studies. Given this sensitivity, it is unlikely that an eligible peer-reviewed study was overlooked, indicating a genuine gap in the published literature. This zero-return should, however, be interpreted alongside two *a priori* eligibility filters. By restricting inclusion to English-language, peer-reviewed sources, the review excluded, by design, grey literature and sport-federation or high-performance testing reports; the sources in which canoeing and sailing monitoring data are most likely to appear. It is possible that relevant monitoring practices in sailing and canoeing are documented in grey literature or high-performance sport reports, which were not captured within the scope of this peer-reviewed review. The finding therefore reflects an absence of eligible peer-reviewed evidence rather than evidence that these athletes are not monitored. In canoeing, the lack of published data highlights a possible research delay in translating standard laboratory protocols into ecologically valid field monitoring tools. Whereas in sailing, this literature gap highlights the unique challenges of collecting data of this nature in this sport. Logistically, sailing takes place across open-water courses, creating possible signal transmission and tracking difficulties not seen in the confined, slightly more predictable environments of for example rowing. Despite advances in technology, it remains a complex task to separate a sailor's actual physical effort or performance from the mechanical movements and speed of the boat itself. Finally, current commercial tracking hardware is unlikely to withstand constant water exposure (often salt water), corrosion, and the risk of capsizing, which could fully submerge the device. Understanding these obstacles explains why research is limited and emphasises the need for the development of specialised technology that can isolate athlete load from boat kinetics.

A profound literature gap identified through this review is the large imbalance of male-derived data and the failure to account for age and sex as confounding variables. It has been well documented that physiological and biomechanical loads differ significantly across sexes and age groups. Therefore, if researchers, sport scientists or coaches apply general baselines to female and youth cohorts, this has a risk of introducing substantial error. Consequently, future research must intentionally evaluate and, where appropriate, design age- and sex-specific monitoring methods to ensure validity. Due to physical and physiological differences associated with age, studies should consider youth athletes on their own and determine whether the same athlete monitoring methods apply to them as to adult athletes. Furthermore, male and female athletes are distinct, and differences in training load between sexes should be considered, as applying male-derived monitoring reference ranges to women risks systematic misclassification of load.

While classifying monitoring methods into tracking, self-report, and performance testing provides a functional framework for mapping the literature, this approach admittedly oversimplifies modern athlete monitoring. In contemporary sports science, these three domains rarely operate in isolation. Instead, modern systems rely on the real-time integration of multiple factors, such as utilising subjective self-reports to contextualise objective tracking data or using daily tracking metrics to adjust the timing of high-intensity training and/or performance testing. Consequently, while this macro-level classification is necessary to integrate the broad landscape of surface water sports literature, it does not fully capture the dynamic, interconnected nature of data integration in these environments. Future research should examine how these monitoring methods overlap within specific sports.

### 4.1 Limitations

This is the first scoping review to map out the methods currently being used in the field of athlete monitoring in non-motorised surface water sports. However, several limitations must be acknowledged. Firstly, this review only considered competitive, high-level athletes performing at a national or international level and did not include any intervention studies. Secondly, this review did not directly compare the different physiological and environmental demands of the various sports. As such, the conclusions are not generalisable to recreational athletes or sports beyond the immediate scope of this review and are limited in their comparisons between monitoring methodologies across surface water sports. In particular, the exclusion of grey literature may disproportionately affect sports such as canoeing and sailing, where monitoring data are more often held in unpublished federation or high-performance settings than in peer-reviewed journals. Thirdly, the inclusion of descriptive, non-analytical observational designs presented challenges in reviewing study quality. Additionally, the authors acknowledge that the search terms used in this review may not have fully captured broader athlete-monitoring constructs, such as readiness, recovery, wellness, and integrated monitoring systems. Future reviews may benefit from incorporating these terms to provide a more comprehensive overview of the field. Lastly, the decision to restrict the search to English-language literature and traditional academic databases was made due to team-capacity, which comprised of English speakers only. Consequently, the potential for language and publication bias cannot be ruled out, as relevant findings documented in other languages or within grey literature sources may have been excluded.

### 4.2 Practical applications

This scoping review shifts the focus from field-based team- and running-based sports to non-motorised surface water sports and aims to provide coaches, athletes, sports scientists and support staff with information relating to the methods used to monitor athletes competing in non-motorised surface water sports. This review highlights that, while wearable technology such as HR and GPS monitors is being used successfully for load monitoring of athletes in non-motorised surface water sports, in practice, until environment-adjusted metrics exist, duration- and HR-based methods should be treated as context-dependent rather than absolute, and practitioners should cross-reference with sRPE.

## 5 Conclusion

The most commonly used methods to monitor load in competitive non-motorised surface water sports within the specific categories include self-report measures (session RPE), tracking measures (duration), and performance testing (HR-based methods). To summarise, the review supports practitioners and researchers in using a mixed-methods approach to monitoring, including both internal and external load variables, such as duration and HR-based methods. Importantly, the unique challenges and constraints of non-motorised surface water sports suggest a need for athlete monitoring methods that account for both dynamic environments and sport-specific biomechanics. Therefore, a generic athlete-monitoring framework is insufficient; understanding the training and competition demands of non-motorised surface-water sports requires a sport-specific approach that explicitly integrates environmental context and potentially specialised mechanical monitoring methods. Additionally, more research is needed on female and youth athletes in the respective non-motorised surface water sports, since these are athletes that are unique and under-represented in the current literature. Finally, more focused systematic review of the existing literature should be conducted on specific surface water sports, to better establish differences related to physiological, biomechanical, perceptual-motor factors.

## Data Availability

The original contributions presented in the study are included in the article/Supplementary Material, further inquiries can be directed to the corresponding author.
