# Supplementary material for: Competition and training load of high-level competitive non-motorised surface water sports athletes: a scoping review of athlete monitoring methodology
Source: Front Sports Act Living. 2026 Jul 28;8:1811408. doi: 10.3389/fspor.2026.1811408 (PMC13457226; doi:10.3389/fspor.2026.1811408)
Supplement: Supplementary file 1 [file Table1.pdf]

Supplementary Appendix 1

Table 1. Search string for corresponding databases

**Database**	**Search string**
Academic Search Premier	(TI("athlete monitoring") OR TI("monitoring methods")) AND (TI("training load") OR TI("internal load") OR TI("subjective") OR TI("external load") OR TI("objective") OR TI("activity profile")) AND (AB("surface water sport" OR "canoe*" OR "kayak*" OR "kiteboard*" OR "paddle*" OR "row*" OR "sail*" OR "stand up paddle" OR "SUP" OR "surf*" OR "windsurf*"))
Google Scholar,	(AB("Athlete monitoring" OR "Monitoring methods") AND ("training load" OR "internal load" OR "subjective" OR "external load" OR "objective" OR "activity profile") AND ("surface water sport" OR "canoe*" OR "kayak*" OR "kiteboarding" OR "paddle*" OR "row*" OR "sail*" OR "stand up paddle*" OR "SUP" OR "surf*" OR "windsurf*"))
SPORTDiscus™	TI("Athlete monitoring" OR "Monitoring methods")<br>AND TI("training load" OR "internal load" OR subjective OR "external load" OR objective OR "activity profile")<br>AND AB("surface water sport" OR canoe* OR kayak* OR kiteboard* OR paddle* OR row* OR sail* OR "stand up paddle*" OR SUP OR surf* OR windsurf*)
SpringerLink	("Athlete monitoring" OR "Monitoring methods") AND ("training load" OR "internal load" OR "subjective" OR "external load" OR "objective" OR "activity profile") AND ("surface water sport" OR "canoe*" OR "kayak*" OR "kiteboarding" OR "paddle*" OR "row*" OR "sail*" OR "stand up paddle*" OR "SUP" OR "surf*" OR "windsurf*")

TI, search restricted to title, AB, search restricted to abstract (Academic Search Premier syntax); Boolean operators AND / OR were used to combine search terms. All databases were filtered for peer-reviewed, English papers only.
